# Bone marrow chimeric mice reveal a dual role for CD36 in *Plasmodium berghei *ANKA infection

**DOI:** 10.1186/1475-2875-6-32

**Published:** 2007-03-16

**Authors:** Margarida Cunha-Rodrigues, Sílvia Portugal, Maria Febbraio, Maria M Mota

**Affiliations:** 1 Unidade de Malária, Instituto de Medicina Molecular, Faculdade de Medicina da Universidade de Lisboa, Av. Prof. Egas Moniz, 1649-028 Lisboa, Portugal; 2 Instituto Gulbenkian de Ciência, 2781-156 Oeiras, Portugal; 3 Cell Biology, Lerner Research Institute, Cleveland Clinic, Cleveland, Ohio, USA

## Abstract

**Background:**

Adhesion of *Plasmodium*-infected red blood cells (iRBC) to different host cells, ranging from endothelial to red blood cells, is associated to malaria pathology. *In vitro *studies have shown the relevance of CD36 for adhesion phenotypes of *Plasmodium falciparum *iRBC such as sequestration, platelet mediated clumping and non-opsonic uptake of iRBC. Different adhesion phenotypes involve different host cells and are associated with different pathological outcomes of disease. Studies with different human populations with CD36 polymorphisms failed to attribute a clear role to CD36 expression in human malaria. Up to the present, no *in vivo *model has been available to study the relevance of different CD36 adhesion phenotypes to the pathological course of *Plasmodium *infection.

**Methods:**

Using CD36-deficient mice and their control littermates, CD36 bone marrow chimeric mice, expressing CD36 exclusively in haematopoietic cells or in non-haematopoietic cells, were generated. Irradiated CD36^-/- ^and wild type mice were also reconstituted with syngeneic cells to control for the effects of irradiation. The reconstituted mice were infected with *Plasmodium berghei *ANKA and analysed for the development of blood parasitaemia and neurological symptoms.

**Results:**

All mice reconstituted with syngeneic bone marrow cells as well as chimeric mice expressing CD36 exclusively in non-haematopoietic cells died from experimental cerebral malaria between day 6 and 12 after infection. A significant proportion of chimeric mice expressing CD36 only in haematopoietic cells did not die from cerebral malaria.

**Conclusion:**

The analysis of bone marrow chimeric mice reveals a dual role of CD36 in *P. berghei ANKA *infection. Expression of CD36 in haematopoietic cells, most likely macrophages and dendritic cells, has a beneficial effect that is masked in normal mice by adverse effects of CD36 expression in non-haematopoietic cells, most likely endothelial cells.

## Background

Malaria is the consequence of *Plasmodium *infection that occurs in a variety of forms, affecting many organs, with different pathological and clinical features [1]. The mechanisms behind the pathogenesis of the various disease syndromes remain poorly understood. The interactions of infected red blood cells (iRBC) with various cells of the host (often referred to as adhesion phenotypes) are thought to be involved in several features of the disease. The accumulation of iRBC in the microvasculature of various organs is believed to protect the parasite from elimination by the spleen and to be a prominent cause of organ-specific malaria syndromes [2]. Non-opsonic phagocytosis is thought to eliminate parasites in a non-inflammatory manner that is reminiscent of the removal of apoptototic cells [3-8]. Several host cells that are involved in iRBC adhesion phenotypes are also involved in general inflammation processes and immune responses of the host. CD36 is a multiligand scavenger receptor that is expressed by many of these cells such as endothelial cells, platelets, dendritic cells and macrophages [9], as well as on both erythroid precursors [10] and normal erythrocytes [11,12]. Adhesion phenotypes involving CD36 include iRBC binding to endothelial cells [13,14] and platelet mediated clumping [15], both of which contribute to the occlusion of the microvasculature in target organs. Non-opsonic uptake of iRBC contributes to the clearance of parasites and modulation of phagocytic cells [3-8]. Thus, CD36 is involved in a variety of host-parasite interactions that may be protective and harmful to both the host and the parasite (reviewed in [8]). Moreover, studies attempting to associate severe malaria syndromes with CD36 polymorphisms in human populations have yielded conflicting results [16-18]. Thus, the role of CD36 in the pathology of human malaria remains elusive and CD36-mediated adhesion cannot be considered as a validated target for anti-malarial intervention.

In the present work, CD36 bone marrow chimeric mice were generated to dissect and measure the contribution of CD36 expression by resident cells *versus *its role in circulating cells to disease outcome. The exclusive expression of CD36 in haematopoietic cells resulted in 25% protection from experimental cerebral malaria (ECM). In wild type mice this protective effect appears to be counteracted by CD36 expression in non-haematopoietic cells. These results indicate that, to be effective, agents that target CD36-binding for malaria intervention need to be cell-type specific, a requirement that cannot readily be met by present day technologies of drug development.

## Methods

### Mice, parasites and infection

Seven to nine-week old male CD36-deficient mice (C57Bl/6 background) [19] and their wild type littermates were infected with fresh 10^6 ^green fluorescent protein (GFP)-expressing *Plasmodium berghei *ANKA iRBCs [20] by intra-peritoneal (i.p.) injection, after one passage through mice infected from a frozen vial. Peripheral blood parasitaemia was determined by flow cytometry and expressed as percentage of infected red blood cells, as described elsewhere [20]. Mice were observed for cerebral complications, i.e ataxia, paralysis, deviation of the head and convulsions, coma and death.

### CD36 bone marrow chimeric mice

CD36^-/- ^mice were lethally γ-irradiated (900 rads) and reconstituted with CD36^+/+ ^bone marrow cells (CD36^+/+ ^into CD36^-/- ^chimeras) or CD36^-/- ^bone marrow cells (CD36^-/- ^into CD36^+/+^). Reverse chimeric mice were also created by lethally irradiating CD36^+/+ ^mice and reconstituting them with either CD36^-/- ^(CD36^-/- ^into CD36^+/+ ^chimeras) or CD36^+/+ ^bone marrow cells (CD36^+/+ ^into CD36^+/+ ^chimeras). Bone marrow cells from donor mice (6.5 × 10^6^) were injected into each recipient mouse through the retro-orbital venous plexus one to five hours after irradiation. Chimeric animals were allowed to recover for five weeks before infection was initiated as described above. Depletion of circulating cells and reconstitution with donor cells was confirmed in all chimeric mice by PCR of blood genomic DNA. Blood was collected from the tail and DNA extracted using QIAamp DNA Micro Kit (Qiagen). PCR was performed using PCR Master Mix (Promega kit) and a set of three primers that amplify sequence either from the wild type (600 bp) or knockout (750–800 bp) allele. The following oligonucleotides (5'-3') were used for specific amplification: CAGCTCATACATTGCTGTTTATGCATG, GGTACAATCACAGTGTTTTCTACGTGG and CCGCTTCCTCGT GCTTTACGGTATC. Amplification program: 95° 4 min-1 cycle; 94° 1 min, 65° 1 min, 72° 2 min- 30 cycles; 4° ∞.

## Results and discussion

CD36 is expressed by both haematopoietic and non-haematopoietic cells [9-12]. CD36 is not only an important mediator of microvessels obstruction by *Plasmodium falciparum *iRBC, but also required for the non-opsonic phagocytosis of different stages of *P. falciparum *iRBC [4,5,21,22]. No consensus has been reached regarding the role of CD36 in the development of severe malaria syndromes in humans [8,23]. Since rodent malaria iRBCs also bind CD36 [24,25], mouse models may be used to study the requirement of CD36 in various features of *Plasmodium *infection [26]. Like in humans, *Plasmodium *infection of mice results in different pathologies that depend on the genomes of both the parasite and the host [26,27]. Previous work has shown that CD36 is essential for the accumulation of *P. berghei *ANKA iRBC in the lungs and adipose tissues, but has no role in the development of parasitaemia and the lethal course of ECM [25].

**Figure 1:**
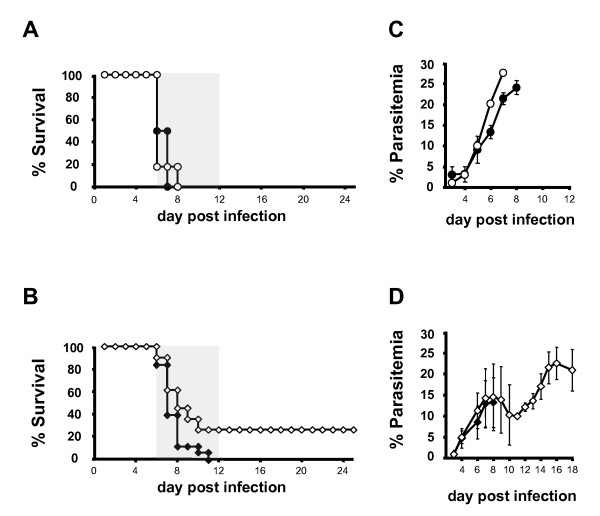
**Bone marrow chimeric mice with CD36 expression confined to lethal radiation-sensitive cells are partially protected from CM**. (A) Cumulative survival and parasitaemias (C) of CD36^+/+ ^into CD36^+/+ ^(n = 11) and CD36^-/- ^into CD36^+/+ ^(n = 11) through the course of *P. berghei *ANKA infection. (B) Cumulative survival and parasitaemias (D) of CD36^+/+ ^into CD36^-/- ^(n = 31) and CD36^-/- ^into CD36^-/- ^(n = 18) through the course of *P. berghei *ANKA infection. Survival curves represent the summary of four of independent experiments. Parasitaemia curves are from one representative experiment out of the four performed. In two independent experiments, CD36^+/+^bm^CD36-/- ^and CD36^-/-^bm^CD36+/+ ^were generated and infected at same time. LOG-RANK Test: *P *< .001 for curve (B). ● CD36^+/+ ^into CD36^+/+^, ○ CD36^-/- ^into CD36^+/+^, ◆ CD36^-/- ^into CD36^-/-^, ◇ CD36+/+ into CD36^-/-^. Gray area represents the time window for ECM development.

To further define the role of CD36 expression in circulating cells such as macrophages and DCs, CD36 chimeric mice were generated by bone marrow transplantation. To control for irradiation effects CD36^-/- ^mice and wild-type mice were also reconstituted with syngeneic bone marrow cells. Five weeks after reconstitution, chimeras showed more than 90% chimerism, as determined by blood genomic DNA PCR (data not shown). The four groups of reconstituted mice were then infected with *P. berghei *ANKA parasites that induce ECM in mice with the C57BL6 background. The two groups of mice reconstituted with syngeneic bone marrow cells (Figure 1A and 1B) and CD36^-/- ^into CD36^+/+ ^chimeras (Figure 1A) died six to 12 days after infection with neurological symptoms like previously observed for non-irradiated CD36^-/- ^and wild type C57BL/6 mice. However, a significant proportion (25.8 ± 9.3 %, n = 31) of chimeras expressing CD36 exclusively in haematopoietic cells, but not in other cells (CD36^+/+ ^into CD36^-/- ^chimeras) did not develop ECM (Figure 1B). These mice survived ECM and died later with higher parasitaemias (Figure 1D). No differences were observed between each chimeric group and its syngenic control in the development of peripheral blood parasitaemia (Figure 1C and 1D). These findings suggest a small, but significant beneficial role for CD36 when expressed only in haematopoietic cells and an adverse effect when expressed elsewhere.

CD36-mediated adhesion phenotypes involving haematopoietic cells include non-opsonic phagoytosis of iRBC by macrophages and dendritic cells and platelet mediated clumping. The later contributes to the obstruction of the microvasculature [15,28] and thus it is unlikely to be responsible for the beneficial effect of CD36 expression in haematopoietic cells that is apparent in the CD36^+/+ ^into CD36^-/- ^chimeras. Whether additional abrogation of CD36 expression in platelets of CD36^+/+ ^into CD36^-/- ^chimeras would lead to a higher protection still remains to be elucidated.

The present study provides the first *in vivo *evidence, albeit indirect, that non-opsonic phagocytosis of iRBC by macrophages and dendritic cells may have a beneficial effect in malaria infections and reduce the risk of development of ECM. In non-chimeric animals, as it might be in humans, this beneficial effect of CD36 is apparently offset, by adverse effects of CD36 expression in non-haematopoietic cells, such as endothelial cells. Our results suggest that therapeutic effects could only be achieved by either up-regulating CD36 expression in macrophages and dendritic cells and/or by downregulating CD36 expression by endothelial cells. A recent study has shown that CD36 expression and phagocytosis of iRBC could be selectively up-regulated by agonists of the peroxisome proliferator activated receptor γ (PPARγ) such as troglitazone [29]. Such selective up-regulation of CD36 in macrophages and dendritic cells may shift the balance towards the beneficial effects of CD36. However, this approach is not likely to have a significant effect in endemic areas where the populations have developed anti-*Plasmodium *antibodies and are thus less dependent on non-opsonic phagocytosis.

## Conclusion

Although clear differences between malaria infections in humans and mice exist, certain aspects of the infection are comparable [26]. CD36 expression and CD36-mediated adhesion of iRBCs to the microvasculature, for example, has been shown not only for *P. falciparum*, but also for the rodent parasites *Plasmodium chabaudi *[24] and *P. berghei *[25]. Studies on human populations polymorphisms for CD36 failed to define a clear role for CD36 in severe malaria syndromes [16-18]. The present study with bone marrow chimeric mice revealed both beneficial and adverse effects of CD36 in infections with *P. berghei *ANKA. Taken together with observations in humans, this study in mice indicates that CD36 is not a straightforward anti-malarial drug target and does not support further efforts to develop drugs that indiscriminately block all CD36 functions.

## Authors' contributions

MCR performed all experimental work, with exception of mice genotyping, and participated both in the study design and manuscript drafting. SP performed mice genotyping, RNA extractions and helped on chimeric mice experiments. MF participated in the study design and manuscript drafting. MMM conceived the study and participated in its design and coordination as well as in manuscript drafting. MMM supervises MCR and SP. All authors read and approved the final manuscript.
